# Fat-Free Mass and the Balance Error Scoring System Predict an Appropriate Maximal Load in the Unilateral Farmer’s Walk

**DOI:** 10.3390/sports6040166

**Published:** 2018-12-08

**Authors:** Michael E. Holmstrup, Michael A. Kelley, Kyla R. Calhoun, Caleb L. Kiess

**Affiliations:** Department of Exercise and Rehabilitative Sciences, Slippery Rock University, 337 Patterson Hall, Slippery Rock, PA 16057, USA; mak101923@gmail.com (M.A.K.); krc1008@sru.edu (K.R.C.); clk1010@sru.edu (C.L.K.)

**Keywords:** asymmetrical, balance, body composition, loaded carrying, regression

## Abstract

This study quantified and compared unilateral farmer’s walk (UFW) performance in recreationally active males and females, and determined if additional variables allowed for the prediction of a maximal safe load. Anthropometric (height, body weight (BW), body mass index, body fat percentage, fat-free mass (FFM), and fat mass), muscular endurance (maximal duration side bridge), and balance (Balance Error Scoring System (BESS)) tests were completed. Participants performed a series of 20 s UFW trials (non-dominant side) at a cadence of 66 beats/min. The initial load was 10% of BW and increased by 10% per trial until deviations in spinal alignment or compromised gait patterns were noted, and the series was terminated. The highest load carried before technical failure was recorded. Descriptive and comparative statistics and a stepwise linear regression analysis were utilized to determine relationships between UFW performance and anthropometric, muscular endurance, and balance tests. Males (N = 25) were significantly taller (177.3 ± 6.7 vs. 164.7 ± 7.2 cm, *p* < 0.05), heavier (81.7 ± 7.0 vs. 62.0 ± 9.4 kg, *p* < 0.05), and leaner (14.4 ± 4.4 vs. 22.4 ± 4.8%, *p* < 0.05) than females (N = 26). Further, males had a higher amount of FFM (*p* < 0.05) than females. The males (52.2 ± 9.0, 64% BW) carried a higher average UFW load than the females (32.5 ± 7.1 kg, 53% BW, *p* < 0.05). FFM was strongly predictive of UFW load (load = −9.88876 + 0.88679 × (FFM); r^2^ = 0.774, *p* < 0.0001). The addition of the BESS test further increased the accuracy of the prediction equation (r^2^ = 0.800, *p* < 0.0001). There are differences in UFW performance ability between males and females. As our method does not account for all potential confounding variables, the use of these equations should be combined with technique analysis and participant feedback to ensure an appropriate workload.

## 1. Introduction 

Loaded carrying has gained attention as a physical test and resistance training method through the improved visibility and popularity of strongman competitions. In general, loaded carrying may be a supplementary method of enhancing the outcomes of traditional resistance training programs [1]. Further, loaded carrying may have direct application to activities of daily living and occupational tasks including those found in military, industrial, and agriculture settings [2,3]. There are several distinct variations of loaded carrying, such as “weight in the hands” or “weight on the body”, which involve altering the orientation of load about the individual. These variations include farmer’s walks (weight in hands), yoke walk (weight across upper back), rack carries (weight across upper chest), Zercher carries (weight in the crook of elbows), and overhead carries (weight in hands, arms extended overhead). Individuals can also perform several of these variations unilaterally. Researchers have begun the work of characterizing the biomechanical and physiological aspects of loaded carrying [1,2,3,4,5,6,7,8], though many considerations, including evidence-based programming recommendations for loaded carrying as a training methodology, remain unclear.

The unilateral farmer’s walk (UFW), or suitcase carry, is a functional, multi-planar activity that involves the coordinated effort of core and hip musculature to maintain posture about the frontal, sagittal, and transverse planes. Further, the oscillatory nature of the UFW during the act of walking challenges the individual to stabilize the thorax on a rotating pelvis [8], calling upon a coordinated, functional effort. Unilateral lifting and carrying tasks result in asymmetrical loading of the spine [2,8] and have been shown to result in increased lateral bending of the lumbar region [7] and increases in spinal compression and lateral shear forces [8] when compared to symmetrical activities. McGill et al. similarly reported that the act of carrying one 30 kg kettlebell in each hand (60 kg total) resulted in markedly lower measures of spinal compression that carrying one 30 kg kettlebell unilaterally [3]. In light of these findings, which warrant a cautious approach when dealing with asymmetrical loads, individuals from the clinical/rehabilitative to strength and conditioning disciplines should also recognize the unique challenge and opportunities for improvement provided by unilateral loading.

The UFW exercise is simple to perform, requiring the participant to carry an implement on one side (like a suitcase) during a controlled walk. Despite this simplicity, the proper management of this offset load during the UFW requires symmetrical and asymmetrical muscle recruitment of the core musculature [9,10], a high demand for trunk muscle co-contraction and synchronicity [11,12], and balance [13]. An elegant study by Daneels et al. characterized the patterns of global (i.e., gluteus maximus, rectus abdominis, external oblique, iliocostalis lumborum pars thoracis) and local muscles (i.e., lumbar multifidus, transversus abdominis, internal oblique) involved with spinal stabilization during asymmetrical lifting tasks, not unlike the UFW [10]. Interestingly, these muscles fire in different patterns (e.g., global muscles fire asymmetrically, favoring the ipsilateral or contralateral side depending on the role of the muscle; local muscles fire symmetrically) during an asymmetrical task. The additional activation of the rectus abdominis, external obliques, latissimus dorsi, rectus femoris, biceps femoris, and gluteus medius during the locomotive portion of the UFW task [1,14] add to the challenge of this activity. Previous work has demonstrated the importance the gluteus medius and its direct effect on hip stability during loaded carrying, noting that weakness in this particular muscle may expose the participant to poor hip alignment during task performance [15,16]. The importance of the gluteus medius may become even more marked when unilateral loading is present [16]. Therefore, the UFW, like other asymmetrical lifting tasks, may present with an increased level of spinal and hip compression [3,8,15], lateral shear [8], and potential for poor hip alignment [15], though also the potential trade-off of unique single and multiple muscle recruitment patterns [10,16]. Currently, there is no guideline present for safe load prescription in loaded carrying, and specifically the UFW, to govern this careful balance.

As previous work has demonstrated, as muscle activation of at least 50% of the maximal voluntary isometric contraction is necessary to elicit strength adaptations in the muscles that provide stability to the maintenance of a neutral spine, it may not be optimal to simply choose a “light” load for the UFW and progress over time. Similarly, selecting a “heavy” load might have potential implications related to injury risk. The subjective nature of what determines a coach’s appropriate load recommendation is also a potential cause for concern (i.e., what is “heavy” or “light” may vary between practitioners). The lower back is likely more susceptible to injury (e.g., intervertebral discs, core and low back musculature, and connective tissue) with a UFW compared to symmetrical carrying task [3,8].

Thus, the purpose of the present study was to observe and compare several anthropometric and fitness measures to maximal load performance in the UFW to determine recommendations for appropriate prescription of this unique, challenging exercise. Based on previous work by Beck et al. who examined the duration of loaded carrying performance, anthropometric measurements were assessed as indicators of maximal load performance [4]. Likewise, the side bridge test, a valid and reliable assessment of core endurance (particularly lateral stabilization), was chosen as a potential predictor of maximal UFW load due to similarities in muscle recruitment between these tasks [17]. Finally, the Balance Error Scoring System (BESS) was included in the analysis as the balance and postural stability characteristics measured may be able to illuminate differences in the center of pressure, postural sway, and core stability required during asymmetrical load carriage across our participants [13,18,19]. As there have been sex differences noted in the performance of both the side bridge test [17] and BESS test [20], a comparison of male and female maximal load performance in the UFW was also undertaken. It was hypothesized that a maximal duration side bridge and BESS test, which may both present a robust challenge to the lateral stabilizers of the core musculature, would predict the maximal safe load performed in the UFW. Moreover, males were expected to demonstrate a higher maximal safe load in the UFW than their female counterparts.

## 2. Materials and Methods

### 2.1. Participants

Fifty-one recreationally active individuals between the ages of 18 and 45 were recruited. All of the study procedures were approved by the Slippery Rock University Institutional Review Board (2016-056-27-B). Participants were required to complete an introductory-level resistance training course prior to inclusion in the study to provide a baseline of technical proficiency across basic movement patterns. Inclusion requirements did not include previous experience with the UFW. Participants were required to complete a health history form, and individuals who did not fit the classification of low risk [21] or who had current musculoskeletal injuries were excluded from the study.

### 2.2. Procedures

Each participant attended two research sessions. Before testing, each participant received pre-test instructions for both testing days. The investigators asked participants to maintain normal sleep patterns and refrain from structured exercise for at least 24 h before each visit. Individuals were asked to wear comfortable exercise clothing to both sessions, which included a sports bra for female participants. Participants were asked to consume their normal food and supplements on the night before and morning of the testing days. Furthermore, participants were asked to maintain consistent hydration for the testing days in line with manufacturer recommendations for body composition analysis (including voiding the bladder within 30 min of body composition testing). At the first data collection session, informed consent and video consent were obtained, and subjects completed forms that outlined their health history. Once cleared for participation, height and weight were measured using a standard doctor’s scale (Chicago model, Continental Scale Works, Chicago, IL, USA). Following specified protocols, a Body Logic Body Fat Analyzer (Omron Healthcare Inc., Vernon Hills, IL, USA) was used to estimate body fat percentage via hand-to-hand bioelectrical impedance analysis (BIA). When used in accordance with protocols, the validity of the Body Logic Analyzer has been compared favorably to hydrostatic weighing (0.91 for males, 0.83 for females; 18–55 years, 7–42.8% body fat) and deemed suitable for use with adults in the present demographic [22]. Fat-free mass (FFM) was calculated by the following equation:

FFM = ((100 − body fat percentage)/100) × body weight (kg)

During both visits, anatomical locations were marked on each participant before the initiation of exercise testing to mark the location of the cervical, thoracic, and lumbar spine during video recording. A small, round sticker was placed in the center of each participant’s chin to indicate the level of the cervical spine. Participants were asked to remove their shirts to mark the location of their xiphoid process with a surgical marker, estimating the level of the thoracic spine. Finally, the region of the lumbar spine was approximated by the visual identification of the umbilicus.

After marking, participants were required to hold an isometric side bridge. This side bridge was completed on the non-dominant side, as conservative unilateral exercise programming would likely prescribe load and volume in relation to the capacity of the weaker side of the body [23]. During the side bridge, each participant maintained a 90° angle between their support arm and their torso. Each bridge was recorded using an iPad device (iPad Air, Apple Inc., Cupertino, CA, USA), and the Hudl Technique app (Agile Sport Technologies, Lincoln, NE, USA) was used to analyze the precise time that proper form (i.e., alignment of the cervical, thoracic, and lumbar spine) was broken. One investigator analyzed the duration of each side bridge. This side bridge test has demonstrated strong reliability (0.97–0.99) in previous studies [17,19].

Following a five-minute rest period, each participant proceeded to the BESS (Balance Error Scoring System) test. During the BESS test, which has been shown to have moderate to high criterion-related validity, good content validity, and correlate with balance testing devices [18], each participant held three separate positions for twenty seconds (double leg, single leg, and tandem) on two different surfaces (standard and foam (AIREX Balance Pad, AIREX AG, Sins, Switzerland); 6 positions total). The foam pad was used to create an unstable surface and increase the difficulty of each position. With eyes closed, participants were required to stand with their hands on their hips for each 20 s interval. Each testing increment began when the participant assumed the proper test position and closed their eyes. The total number of errors across all six positions (e.g., opening eyes, stepping out of position, deviation of the hip joint > 30°) was documented. Like the side bridge test, each BESS protocol was recorded using the “Technique” app for subsequent analysis and analyzed by one investigator.

On the second day of testing, no less than 48 h after the first session, each participant reported to the lab and completed a brisk, five-minute walk on a treadmill as a general warm up. Following the warmup and anatomical marking (identical to day one testing), each participant completed a video-recorded, 20 s UFW with 10% of their body weight (BW) in their non-dominant hand in line with previous recommendations regarding unilateral exercise [23]. No instructions were given regarding the positioning of the dominant hand/arm. Calibrated fractional weight plates (Christian’s Fitness Factory, Lancaster, PA, USA) were used to allow precise loading. When values fell in between fractional increments, loads were rounded up to the nearest 0.25 pounds. The initial weight increment (10% BW) was loaded onto a hollow, adjustable Olympic dumbbell handle (Impex Inc., Pomona, CA, USA; 3 pounds), and all subsequent increments (which increased by 10%) were loaded onto a Vulcan Farmer’s Walk Handle (Vulcan Strength Training Systems, Charlotte, NC, USA; 22 pounds). Participants were asked to match their stride rate to a metronome set to a cadence of 66 beats/min to account for variations in stride frequency. There was a 5 min rest in between each trial. Testing was terminated when any of the following conditions was met: (1) lateral flexion of the spine exceeded 8° (as measured by deviation from the cervical, thoracic, or lumbar line generated by the “Technique” app); (2) the participant’s gait became compromised (i.e., dragging the ipsilateral leg, stumbling gait); or (3) volitional fatigue/failure to complete the 20 s UFW. Upon test termination, the previous weight increment (e.g., 50% if the participant failed on the 60% trial) was recorded as the “safe” maximal load.

### 2.3. Statistical Analyses

Descriptive statistics were generated, and an independent sample *t*-test was used to compare the performance of male and female participants. A stepwise linear regression analysis was performed in order to account for the variance in load prediction by the following variables: age, sex, height (cm), weight (kg), body fat percentage, FFM (kg), fat mass (kg), side bridge time (s), and BESS score (total errors). An a-priori p-value was set at *p* < 0.05. In line with published procedures, effect sizes were generated for differences in the performance variables between sexes (Cohen’s d) using means and standard deviations [24], and significant predictors in the regression model (Cohens ʄ^2^) using standardized coefficients of variation [25]. Data were analyzed with SPSS version 21 for Windows (SPSS Inc, Chicago, IL, USA).

## 3. Results

Twenty-six female and twenty-five males completed all of the requirements, and their characteristics can be found in Table 1. Participants self-reported adherence to all of the pre-test instructions on both testing occasions, and there was no attrition. Male participants were significantly taller and heavier than their female counterparts (*p* < 0.05). Further, male participants had a lower average body fat percentage and higher fat-free mass than females (*p* < 0.05).

All participants completed the test battery, and their performance is summarized in Table 2. Though males were able to hold their side bridge hold an average of 17 s longer than females, this difference was not considered statistically significant (*p =* 0.057; Cohen’s d—0.54, medium effect size). Females had fewer total errors (5.7 vs. 9.0; *p* < 0.05; Cohen’s d—0.76, medium effect size) on the BESS test when compared to males. Females were able to carry an average load of 52.7 ± 8.7% BW, while males were able to carry a larger average load of 63.6 ± 8.6% BW (*p* < 0.05; Cohen’s d—2.45, large effect size).

**Figure 1:**
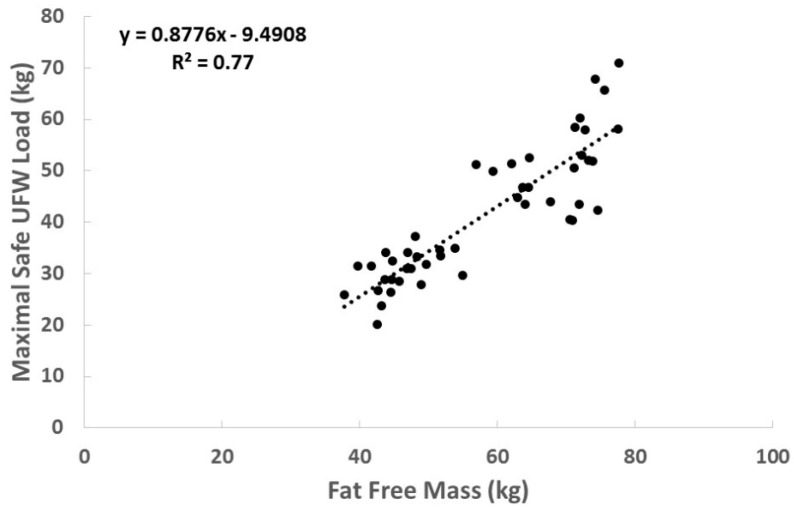
Relationship between FFM and Maximal Safe Load in the UFW. Scatterplot of data comparing FFM (kg) to conservative UFW load carried (n = 51). The line of best fit is presented. The equation for this line and its associated coefficient of determination are located in the upper left corner.

Stepwise linear regression analysis revealed that FFM strongly predicted the maximal safe load carried by participants (r^2^ = 0.77, *p* < 0.0001; Cohen’s ʄ^2^—3.42, large effect size; Figure 1). A maximal safe load for the UFW can be predicted by the following equation:

UFW load = (0.877 × FFM) − 9.491

Further, the addition of the BESS test to the regression model further increased the accuracy of the prediction equation (r^2^ = 0.80, *p* < 0.0001; additional Cohen’s ʄ^2^—0.21, medium effect size). When the BESS test can be included as a component of fitness testing, the following equation can predict a maximal safe load for the UFW:

UFW load = (0.819 × FFM) + (0.482 × BESS) − 9.411

## 4. Discussion 

To the authors’ knowledge, the present study is the first to present safe maximal loading parameters for the UFW. FFM, as well as balance and postural stability (as assessed by the BESS test), were robust predictors of a safe maximal workload for the UFW task. The equations provided here can give the resistance training professional or participant a practical, evidence-based approach to choosing a technically acceptable and effective load for the UFW.

McGill et al. demonstrated that healthy, young males had a greater potential to maintain the side bridge position than females [17]. The findings of our investigation concur with McGill, as our male participants were able to maintain the side bridge position for approximately 18–19% longer than their female peers. Moreover, our average side bridge durations were within 7.5% and 5.2% of the values reported by McGill for males and females, respectively.

Performance on the BESS test in our sample did not follow the trends of previously published research. In contrast to Iverson and Koehle [20] who reported that men did slightly better than women on the BESS, females in our study performed markedly better than males. It is likely that the age of our cohort, which was at the very low end (e.g., female average = 19.7 years) of their 20–69-year old cohort (average age—49.5 ± 10.8 years), may have affected this outcome. Previous work supports this notion, as additional errors in the BESS test are associated with advancing age (with 50 years indicated as a transitive cut off) [26]. Additionally, though we had one researcher quantify the total number of errors during our recorded BESS tests, this scoring system has been shown to become more reliable with three total trials [27].

This study quantified the safe limits of the UFW and provides evidence-based guidelines for the safe performance of this unique exercise. As safety was a primary concern for this study, there were several precautions in place to allow participants to test the limits of their UFW. While researchers visually evaluated each UFW performance in real time to watch for loss of the neutral spine position, video analysis was undertaken during five-minute rest periods in between trials when form was questionable. The presence of lateral flexion or bending during the UFW was of particular interest to the researchers [7]. As Fujimori et al. [28] have presented mean maximal ranges of motion of ~16° between the lumbar (L1) and thoracic (T1) region of the spine, we conservatively determined that half of that range of motion (8°) was the maximal allowable deviation during a safe UFW in our study. Further, subjects were asked to terminate their UFW if their gait was compromised during the task. As each UFW task was completed to the cadence of an intentionally slow metronome beat (66 bpm), it is likely that the loads predicted by our equations may be more manageable when individuals are given the opportunity to walk at their own pace to cover a similar distance.

The most significant predictor of load capacity in our regression model was FFM. This finding is in line with the work of Beck et al. who determined that lean mass was associated with stretcher, jerry can, and kettlebell carries [4]. The carries in Beck et al.’s study were performed at submaximal loads over maximal distances, and the authors’ concluded that training programs designed to address muscle hypertrophy would potentially have a beneficial effect on individuals who were tasked with occupational carries [4]. The current investigation also linked increased FFM with an increased maximal safe load in the UFW. While differences in FFM across various body segments were not addressed in the present study, it is likely that an increase in the propulsive force of the lower body, similar to that shown by Beck et al. [4], may explain some of the predictive nature of FFM on UFW performance. However, as an overall measure of FFM was measured in the present cohort, other factors including overall force production capability, differences in muscle fiber type characteristics, or grip strength may have contributed to differences in UFW performance.

A few limitations must be taken into consideration when interpreting the findings of the present study. Importantly, due to no single investigator being available for all subjects, body fat percentage (and therefore FFM) was estimated with the use of a commercially-available BIA device (Omron Healthcare Inc., Vernon Hills, IL, USA). Hand-to-hand analysis has been shown to have similar reliability as hand-to-foot analysis and has been deemed practical for the assessment of FFM [29], though it may not be the most valid method of body fat assessment. There is likely some overestimation of FFM with hand-to-hand bioelectrical impedance analysis [30]. Ultimately, logical support for our decision to use this means of measurement was practical applicability. Hand-to-hand devices do not require any specialized skills to operate and are commonplace in many fitness centers. Further, the use of the presented equations can only be applied using the handheld BIA method. The findings of this study should only be applied to healthy individuals. All of the participants were free from musculoskeletal issues, and therefore these equations may not be suitable for use in rehabilitation scenarios. Finally, while we had a consistent, quantifiable metric for the loss of neutral spine for our test termination criteria (8° or more of lateral flexion), there was not as consistent of a criterion for compromised gait. In the instances where compromised gait resulted in the termination of the test, a consensus was reached amongst the researchers (and the prior UFW trial was recorded as the maximal safe load). Indeed, this is comparable to when strength and conditioning professionals make a judgment call regarding a technical deficit during a training session.

## 5. Conclusions

In conclusion, loaded carrying is a popular means of enhancing resistance training sessions for sport, strength, and activities of daily living, aside from being an important requirement in many occupational settings. A maximal safe load for the UFW can be predicted by FFM, and this prediction can be further improved by the addition of the BESS test. Future work will be needed to quantify the optimal volume and frequency recommendations for the UFW, and intensity, volume, and frequency recommendations for the loaded carry movement pattern in general.

## Figures and Tables

**Table 1:** Summary of participant characteristics.

Subjects	N	Age (years)	Height (cm)	Weight (kg)	BF ^a^ (%)	FFM ^b^ (kg)
Female	26	19.7 ± 1.1	164.7 ± 7.2	62.0 ± 9.4	22.4 ± 4.8	47.9 ± 6.3
Male	25	21.8 ± 5.2	177.3 ± 6.7 *	81.7 ± 7.0 *	14.4 ± 4.4 *	69.8 ± 5.2 *

All data are reported as mean ± standard deviation. ^a^ BF—body fat percentage, ^b^ FFM—Fat-free mass. * Significantly different from female participants, *p* < 0.05.

**Table 2:** Summary of participant performance.

Subjects	N	Plank (s)	BESS ^a^ (Total Errors)	Load (kg)
Female	26	73.0 ± 27.7	5.7 ± 3.7	32.5 ± 7.1
Male	25	89.6 ± 33.0	9.0 ± 4.9 *	52.0 ± 8.7 *

All data are reported as mean ± standard deviation. ^a^ BESS—Balance Error Scoring System. * Significantly different from female participants, *p* < 0.05.
